# Genetic Polymorphism in Angiotensinogen and Its Association with Cardiometabolic Diseases

**DOI:** 10.3390/metabo12121291

**Published:** 2022-12-19

**Authors:** Momina Shahid, Kanwal Rehman, Muhammad Sajid Hamid Akash, Shaleem Suhail, Shagufta Kamal, Muhammad Imran, Mohammed A. Assiri

**Affiliations:** 1 Department of Pharmaceutical Chemistry, Government College University, Faisalabad 38000, Pakistan; 2 Department of Pharmacy, The Women University, Multan 60000, Pakistan; 3 Department of Pharmacy, University of Chenab, Gujrat 50700, Pakistan; 4 Department of Biochemistry, Government College University, Faisalabad 38000, Pakistan; 5 Research Center for Advanced Materials Science (RCAMS), King Khalid University, Abha 61514, Saudi Arabia; 6 Department of Chemistry, Faculty of Science, King Khalid University, Abha 61413, Saudi Arabia

**Keywords:** AGT polymorphism, M235T, T174M, G-217A, A-6G, A-20C, G-152A, associated diseases

## Abstract

Angiotensinogen (AGT) is one of the most significant enzymes of the renin-angiotensin-aldosterone system (RAAS) which is involved in the regulation and maintenance of blood pressure. AGT is involved in the production of angiotensin I which is then converted into angiotensin II that leads to renal homeostasis. However, various genetic polymorphisms in AGT have been discovered in recent times which have shown an association with various diseases. Genetic polymorphism increases the level of circulating AGT in blood which exaggerates the effects produced by AGT. The associated diseases occur due to various effects produced by increased AGT levels. Several cardiovascular diseases including myocardial infarction, coronary heart disease, heart failure, hypertrophy, etc. are associated with AGT polymorphism. Other diseases such as depression, obesity, diabetic nephropathy, pre-eclampsia, and liver injury are also associated with some variants of AGT gene. The most common variants of AGT polymorphism are M235T and T174M. The two variants are associated with many diseases. Some other variants such as G-217A, A-6G, A-20C and G-152A, are also present but they are not as significant as that of M235T and T174M variants. These variants increase the level of circulating AGT and are associated with prevalence of different diseases. These diseases occur through various pathological pathways, but the initial reason remains the same, i.e., increased level of AGT in the blood. In this article, we have majorly focused on how genetic polymorphism of different variants of AGT gene is associated with the prevalence of different diseases.

## 1. Introduction

Genetic polymorphism is the most common type of genetic variation identified in the human genome. It is characterized by the presence of two or more alternative allele forms in any individual’s genome that result in various phenotypes in the same population [1]. Genetic polymorphism occurs as a result of inconsistencies in DNA sequence among the populations. There are many other origins of genetic diversity, including single nucleotide polymorphism, repetitive DNA and RNA patterns, the presence or absence of particular nucleotide sequences, and the interchange of genetic material. Additionally, the genetic mutation causes polymorphism, which results from modifications in the nucleotide sequence [2].

Mutations are the fundamental processes that give rise to almost all genetic variants. Many scientists concur that mutation is the irreversible sequence variation in DNA, which in essence encompasses all the variations occurring in the human genome, whether they are spontaneous or not [3]. Mutation can lead to epigenetic modifications by the alteration of DNA methylation patterns or the change of particular histone types, which have major effects on local chromatin structure [4]. Acetylation, methylation, phosphorylation, and SUMOylation are the few histone modifications. DNA methylation primarily occurs in CpG (5′-C-phosphate-G-3′) dinucleotide at position 5 of cytosine residues to produce 5-methyldeoxycytidine [5]. Genetic variations in the genome are significantly influenced by the interaction of numerous environmental factors [6]. Gene mutation occurs at the single nucleotide level is known as single nucleotide polymorphism. It occurs due to the changes in amino acid sequences, which affect the transcription, intrinsic termination, and factor-dependent termination, this also results in altered enzyme activity [7].

**Figure 1:**
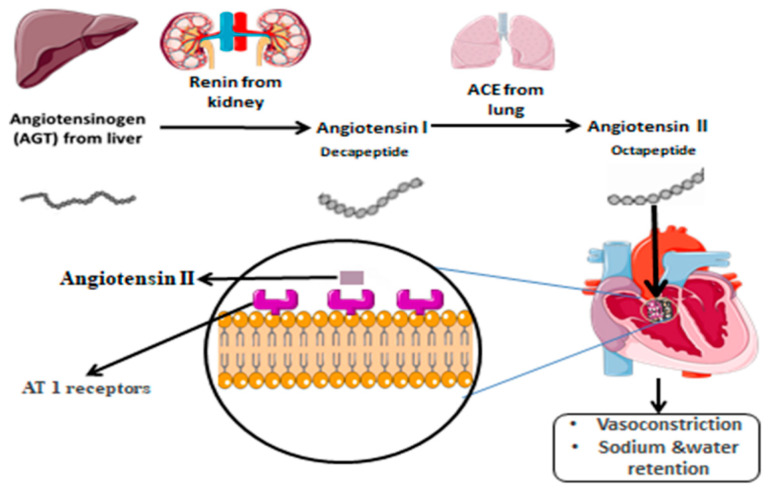
Graphical representation of the mechanism of action of AGT that is involved in the renin-angiotensin system.

The human angiotensinogen (AGT) gene is a member of serpins gene superfamily, which is 12 kb in length with 5 exons and four introns located on chromosome 1(1q42-q43) [8]. It is found in numerous tissues including liver, adipose tissue, heart, vascular wall, brain, kidney, and is equally wide in its cell specificity [9]. All angiotensin peptides originate only from AGT. Human AGT contains 485 amino acids with 33 amino acid signal peptides. Angiotensin I (Ang I), which is the source of a variety of active angiotensin peptides, is produced by renin by cleaving the 10 amino acids from N-terminal [10]. The N-terminal amino acids of mature AGT produced by hepatocytes are cleaved intravenously by the renin enzyme, a component of Renin-Angiotensin-Aldosterone System (RAAS). Renin, which is secreted by juxtaglomerular cells, first cleaves itself to produce angiotensin-I decapeptide and then angiotensin-converting enzyme (ACE) produces the angiotensin II octapeptide. The RAAS cascade’s rate-limiting stage, which regulates plasma AGT levels and is essential for blood pressure maintenance, is the renin-AGT enzymatic reaction [11]. To perform its functions angiotensin II binds G-protein-coupled receptors on cell membranes. Kidneys express two different types of angiotensin-II receptors, which have 359 and 363 amino acids, respectively, and share 30% of their amino acid sequences. These receptors are known as AT1R and AT2R, and are found in brain, heart, endothelium of adipose tissue, adrenal glands, vascular smooth muscle, and kidneys [12]. Angiotensin II’s octa-peptide, required for interaction with its receptors, is the main peptide of RAAS. The human body expresses the AT1R angiotensin II receptor. AT1Rs are one of the key determinants of angiotensin II, which regulates the electrolyte balance and blood pressure [13], as shown in Figure 1.

**Figure 2:**
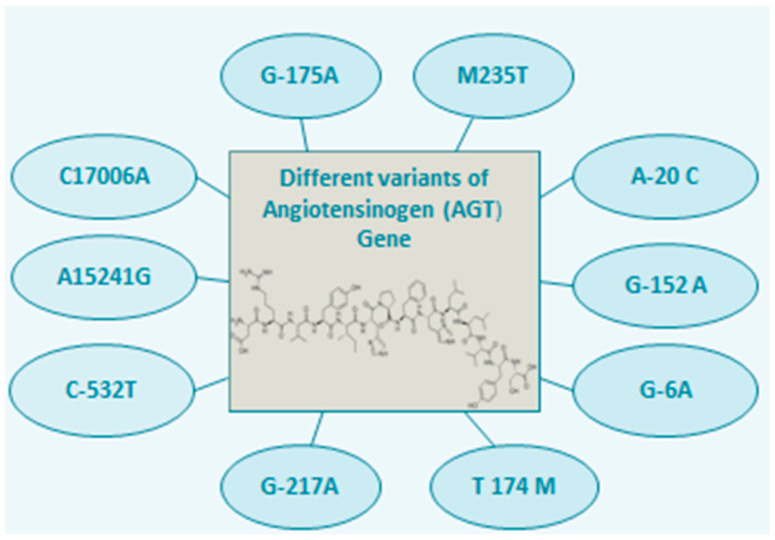
Graphical representation of different variants of the AGT gene that are linked to various diseases.

The promoter and neighboring elements are the regions of AGT that have been identified as significant regulators of AGT expression, which are positioned roughly at 1.2 kb upstream of exon 1, and an enhancer was found in 3′ surrounding areas immediately following the second polyadenylation site [14]. Polymorphisms in the promoter region are essential because they may affect the strength of AGT promoter, which in turn may impact the levels of AGT and angiotensin II [15]. Different variants of AGT gene have been identified including AGT T174M, M235T, G-152A, G-6A, G-175A, A-20 C, C17006A and A15241G [16,17,18,19] as shown in Figure 2.

AGT M235T (rs699) and T174M (rs4762) are the two most common and significant polymorphic forms of AGT. The single nucleotide polymorphisms of AGT T174M (rs4762) and AGT G-6A (rs5051) have been linked to hypertension in Europe and Asia. They involve a change from guanine to adenine in exon 2 of the gene and a subsequent function switch from threonine (T) to methionine (M) at codon 207 (G/A, T207M at position 174), and have been correlated with increased plasma AGT. AGT rs4762 has also been connected to an elevated risk of CKD development. AGT rs4762 has been associated with early onset of hypertension and increased systolic blood pressure (SBP) in the Hutterian Brethren and Japanese populations. GT rs5051, on the other hand, has been strongly linked to hypertension in the people from US, France, and Mexico [20,21]. Angiotensinogen levels have been discovered to be correlated with T174M polymorphism [22]. The single nucleotide polymorphism of AGT M235T (rs699) has been linked to the development of coronary artery diseases (CAD) [23]. It involves the replacement of T nucleotide at position 704 of the second exon by the C nucleotide, resulting in the conversion of amino acid at position 235 from methionine (M) into threonine (T) [24]. The T allele of M235T most likely increases the risk of CAD in the presence of hypercholesterolemia because of the pleiotropic and proatherosclerotic characteristics of hypercholesterolemia and angiotensinogen derivatives [25]. This review highlights the genetic polymorphism of different variants of AGT and its associated diseases.

## 2. AGT Polymorphism and Associated Diseases

### 2.1. High Altitude Polycythemia

High altitude polycythemia (HAPC) is a disease that refers to living long term in the plateau of a hypoxic environment leading to hyperplasia of red blood cells. Symptoms mostly caused by HAPC are vasodilation, difficulty in breathing, dizziness, and headache [26]. The AGT M235T polymorphism was related to oxygen saturation which is associated with the development of HPAC. A case–control study suggested a correlation between the polymorphism of AGT M235T and development of HPAC in Chinese Han and Tibetan populations [27]. RAAS is essential for controlling the blood pressure as well as maintaining the fluid balance in circulatory system. Additionally, it has a variety of effects on target tissues. An enzyme cascade including proteases renin and ACE produces angiotensin II, an important factor in modulating the blood pressure [28]. RAAS activation increases the erythropoiesis and decreases oxygen saturation, mostly through increasing the plasma erythropoietin levels through kidney AT1R leads to development of high-altitude polycythemia [29].

### 2.2. Pre-Eclampsia

Pre-eclampsia is a multifactorial hypertensive disorder usually manifested as a rise in the level of proteinuria. It can cause pregnancy complications such as preterm birth, fetal and maternal mortality, and intrauterine growth restriction of the fetus [30]. AGT is involved in the synthesis of angiotensin and important for the control of blood pressure. AGT gene, found on chromosome 1, is involved in the pathogenesis of pre-eclampsia. The AGT M235T polymorphism increases the level of AGT which affects the spiral arteries in uterus. This is one of the early causes of pre-eclampsia [31,32]. As a sole renin substrate, AGT plays a significant role in the RAAS. The biosynthetic precursor of renin; Pro-renin, is a peptide that blocks AGT from binding to the renin through the pro-renin receptor (PRR). Overexpression of pro-renin receptor by oxidative stress increases the concentrations of angiotensin-II due to increased angiotensin-I breakdown from AGT [33]. The vasoconstrictor effects of angiotensin -II are caused by the production of ROS [34]. RAAS components act on spiral arteries in pregnant women during 1st trimester leads to pregnancy induced vessel remodeling and cause placental implementation and utero-placental ischemia leads to the development of pre-eclampsia [35]. Various studies show the significant association of AGT M235T gene polymorphism and pre-eclampsia [36,37]. Some studies contradict these findings and do not show the association between AGT M235T gene polymorphism and pre-eclampsia [38,39,40].

### 2.3. Obesity

Obesity is an abnormal fat distribution in the visceral parts of the body. This excess fat distribution can result in altered hormonal functions and can also stimulate different mechanisms within the body such as hypertension [41]. Many studies showed a significant association of AGT polymorphism with obesity [42]. Polymorphism of AGT M235T and T174M can cause the conversion of pre-adipocytes to adipocytes and increase the number and volume of adipocytes which leads to obesity [43]. The plasma level of AGT was higher in the adipose tissues among obese patients [44]. All RAAS components, including AGT, renin, angiotensin converting enzyme and angiotensin-II type 1 receptor, are expressed in adipose tissue [45]. Overexpression of AGT leads to increased plasma AGT levels. The level of circulating AGT and adipogenesis are influenced by adipose tissue derived AGT which then affects the lipid metabolizing capacity of adipocyte and leads to obesity [46,47]. The gender specific study showed the significant association of AGT M235T gene polymorphism with obesity in males as compared to that in females among the Tunisian population [48]. Another study shows the significant association of AGT M235T gene polymorphism with obesity in females as compared to that in males among the Poland population [43].

### 2.4. Cardiovascular Diseases

Numerous cardiovascular disorders have been linked to RAAS enzymes. AGT M235T polymorphism plays a vital part in the pathogenesis of many cardiovascular disorders (including myocardial infarction, ischemic heart disease, CAD). Evidence suggests that AGT polymorphism can result in vasoconstriction that leads to the progression of atherosclerosis [49].

AGT, a serum renin glycoprotein substrate that is generated and released in the liver as the precursor of angiotensin, the hormone that serves as part of the systemic blood pressure regulation system, is a crucial component of this system [50]. AGT regulates blood pressure; therefore, modifications to its gene sequence are expected to be crucial in the development of cardiovascular risk features including hypertension and the appearance of coronary artery disease (CAD) [51,52]. Vascular illnesses may start and progress due to abnormalities of the renin–angiotensin–aldosterone system (RAAS). Renin and angiotensinogen (AGT), one of the initial components in this system, interact to create angiotensin I, the precursor hormone of angiotensin II. The plasma concentration of AGT is altered by genetic variations in the AGT gene and may play a part in the development of hypertension, coronary heart disease, and myocardial infarction [53]. Angiotensin II can cause fibrosis and the synthesis of collagen. It can also modify the growth of cardiac fibroblasts [54]. All of this ultimately leads to MI. AGT M235T have shown a positive association with MI among various populations. Several meta-analyses have also confirmed this association, but contrary results can also be seen [55,56]. AGT polymorphism is involved in the development of hypertrophy of the left ventricle of the heart. This hypertrophy occurs due to cell hypertrophy and cell proliferation. This can also occur due to partial myocardial hypertrophy [57]. AGT acts as a precursor for the production of angiotensin-converting enzyme (ACE) II. ACE II produces positive ionotropic, hypertrophic, and apoptotic in the myocardial cells. It also affects the center which is responsible for the control of heart failure and hypertrophy. A higher plasma level of AGT results in the increased production of AGT II which, eventually, leads to hypertrophy [58,59].

### 2.5. Diabetic Nephropathy

Diabetic nephropathy is a chronic complication of diabetes. The renal vasculature is damaged which may result in end-stage renal failure (ESRD) [60]. The RAAS has a role in controlling blood pressure, maintenance of renal homeostasis and salt and water retention. All of these processes contribute to the pathogenesis of diabetic nephropathy [61,62]. Excessive AGT in the plasma can lead to constriction of arterioles in the kidneys. This increases the renal and peripheral resistance causing increased glomerular capillary pressure and proteinuria. All of this leads to endothelial dysfunction and renal damage causing diabetic nephropathy. AGT M235T polymorphism increases the plasma level of AGT which increases the risk for diabetic nephropathy [63,64]. The RAAS system is impacted by the polymorphism of AGT. The polymorphism of AGT M235T leads to the replacement of methionine with threonine amino acid (M235T) and a substitution of the T to C base at 702 nucleotides on exon 2 results in the development of diabetic nephropathy [65]. Various gender specific studies showed the significant association of AGT M235T gene polymorphism with diabetic nephropathy in males as compared to females among various populations [66,67]. However, some studies are also available which contradict these findings and do not show the association between AGT M235T and AGT T174M gene polymorphism and diabetic nephropathy [68,69].

### 2.6. Hashimoto’s Thyroiditis

Hashimoto’s thyroiditis is one of the autoimmune disorders of the thyroid. It is also known as autoimmune thyroid disease (AITD). The clinical manifestation of this disease includes the presence of thyroid antibodies in the blood and the infiltration of the autoreactive lymphocytes arising from the thyroid gland [70]. The metabolic status of the thyroid enzymes affects the enzymes of RAAS. Angiotensin is implicated in the production of immune cells which causes autoimmune neuroinflammation. The Th-1 mediated autoimmune response is also thought to be caused by RAAS [71]. The characteristic of the angiotensinogen gene, AGT, is the T174M at position 174 of the mature protein is the substitution of threonine (T) to methionine (M), which is found in exon 2-coding polymorphism. Angiotensinogen levels have been discovered to be related to the T174M polymorphism. The activity of the protein products produced by particular polymorphic variations of the ACE, AGTR1, and AGT genes alter RAAS activation. However, it is unclear what precise function these polymorphisms play in the pathogenesis of thyroid autoimmune disorders [72].

### 2.7. Essential Hypertension

Essential hypertension is a multifactorial disease and is present globally. A lot of diseases are associated with hypertension, and it has become a fetal risk factor due to its high prevalence worldwide. Apart from environmental factors such as diet and lifestyle, the genetic part in the pathophysiology of hypertension is inevitable [73,74]. Various genetic polymorphisms are seen to be associated with hypertension. The enzymes from RAAS (including AGT) play a vital role in the regulation and control of blood pressure through vasoconstriction and sodium homeostasis. AGT not only produces systemic effects, but several local effects are also produced. These local effects include exocrine, endocrine, and paracrine signaling [75]. The AGT gene is present on the long arm of chromosome 1. The exact location of the gene is at chromosome 1q42-3 having 5 exons and 4 introns. A genomic sequence of 13kb is present [76]. The genetic polymorphism of AGT increases the plasma level of AGT [77]. Many genetic variants of AGT have shown a positive association with hypertension. AGT T174M and AGT M235T are one of the most common variants responsible for hypertension in several populations. However, contrary results are also available [22,78]. AGT G-217A and AGT A-20C have also shown positive association in some populations [16,79]. AGT M235T affects the plasma angiotensinogen levels because threonine is substituted for methionine at codon 235. Hypertension is strongly correlated with the T allele of M235T, which is related to greater plasma AGT [80]. Various case–control studies showed a significant association of AGT M235T polymorphism with essential hypertension in females as compared to that in males among the South Indian population [80]. Another study has also shown a significant association of AGT M235T and AGT T174M polymorphism with essential hypertension in females as compared to males among the Indian population [22]. However, contradicting results are also available regarding the association of AGT M235T, AGT T207M, AGT M268T and AGT A-6G gene polymorphism and essential hypertension among various populations [81,82,83,84].

### 2.8. Liver Cirrhosis

Chronic liver disorders eventually reach the stage after years or decades of progression, known as cirrhosis [85]. Cirrhosis is a progressive form of liver fibrosis that is characterized by hepatic vascular deformation. This compromises communication between hepatic sinusoids and the surrounding liver parenchyma, or hepatocytes, by causing the portal and arterial blood supply to be diverted directly into the hepatic outflow [86]. The general circulatory abnormalities in cirrhosis, such as splanchnic vasodilation, vasoconstriction and hypoperfusion of kidneys, water and salt retention, and increased cardiac output, are intimately associated with hepatic vascular alterations and portal hypertension [87]. Angiotensinogen (AGT), a renin substrate, is broken down by the angiotensin-converting enzyme (ACE), a zinc-dependent metallopeptidase, to produce the decapeptide angiotensin-I (Ang I), which is then converted to angiotensin II (Ang II) [88]. Ang II can activate the hepatic stellate cells (HSCs) which are essential for the development of liver cirrhosis [89]. Ang II can also enhance the production of TGF-1 and TIMP1, increase the proliferation and migration of aHSCs as well as collagen synthesis through autocrine and paracrine mechanisms, and inhibit collagen breakdown, resulting in liver cirrhosis [90].

## 3. Discussion

This review highlights the importance of genetic polymorphism and its association with certain diseases. The genetic based studies help in personalized medication based on genetic and epigenetic modifications. For individuals without poor prognosis variables, it could in fact lessen the side effects of unnecessary therapies [91]. The goal of personalized medication is to identify the interventions that will have the most impact on a patient’s illness course depending on their genetic and molecular make-up as well as environmental and epigenetic ecology [92]. In personalized medicine, each patient is identified by a distinct set of molecular abnormalities, the targets are clearly identified, and developing a therapy plan is expected to produce superior outcomes [93]. The scientific foundation for a personalized treatment plan is using cutting-edge nutrigenomic and nutrigenetic mechanisms to make up for deficiencies, mitigate the effects of genetic predispositions that have been identified, and create epigenetic changes in an effort to affect a particular pathology is provided by genetic polymorphism testing [94]. Personalized therapy could be a major step toward more efficient and secure therapeutic choices [95].

## 4. Conclusions

Genetic polymorphism has recently proved to be a risk factor for many diseases. AGT is one of the key enzymes of RAAS. It is involved in the controlling of blood pressure due to its role in the synthesis of angiotensin I and II. Different variants of AGT are present which affects the concentration of AGT in the blood. AGT M235T and T174M have a significant role in the pathogenesis of several diseases. These two variants are common and associated with a greater number of diseases than any other variant of AGT. Different diseases associated with different variants of AGT have been elaborated in Table 1. AGT M235T is seen to be associated with hypertension, pre-eclampsia, diabetic nephropathy, obesity, depression, and various cardiovascular diseases. Similarly, T174M shows an association with diabetic angiopathy, diabetic nephropathy, pre-eclampsia, hypertension, and different cardiovascular diseases. Liver cirrhosis is a disease that did not show association with M235T and T174M, but it was seen that people with A-6G and A-20C polymorphism showed association with this disease. Even though all these polymorphisms increase the level of AGT in the blood, variants other than M235T and T174M, have not shown an association with a variety of diseases. These polymorphisms differ in their prevalence among different populations where M235T and T174M were seen to be more common.

## Figures and Tables

**Table 1:** Diseases associated with polymorphism of different variants of AGT.

Gene Variant	Associated Diseases	Mechanism (Due to High Serum Level of AGT)	Ref.
AGT T174M	Diabetic Nephropathy	Increased peripheral and renal resistance, oxidative stress and endothelial dysfunction	[96]
	Diabetic Microangiopathy	Altered cell matrix, vasoconstriction and impaired vascular tone regulation	[97]
	Myocardial Infarction	Cardiac fibrosis, collagen synthesis and modified growth of cardiac fibroblast led to the development of atherosclerosis	[98]
	Ischemic Stroke	Activation of vascular cell apoptosis, and vasoconstriction	[99]
	Hoshimoto’s Thyroiditis	Autoimmune neuroinflammation	[72]
	Cerebral Stroke	Neurogenic hypertension	[100,101]
	Coronary Artery Disease	Myocardial hypertrophy, vasoconstriction and fibrosis	[102,103]
	Arterial Fibrillation	Stimulation of mitogen-activated protein kinase pathway	[104,105]
	Pre-eclampsia	Vasoconstriction and dysregulation of salt and water homeostasis	[106,37]
	Coronary Atherosclerosis	Endothelial injury due to oxidative stress (oxidation of NADPH generates the oxidative species which further oxidizes the DNA, lipids and lipoproteins leading to endothelial damage)	[107,108]
	Hypertension	Vasoconstriction and increased vascular resistance	[22,109]
AGT M235T	Pre-eclampsia	Vasoconstriction and dysregulation of salt and water homeostasis	[110]
	Diabetic Nephropathy	Increased peripheral and renal resistance, proteinuria and oxidative stress lead to endothelial damage	[111]
	Acute Ischemic Cardiac Dysfunction	Modification of extracellular matrix and myocytes in the heart	[112]
	Heart Failure	Sodium retention, myocardial fibrosis and vasoconstriction	[113]
	Hypertrophic Cardiomyopathy	Angiotensin II exerts positive inotropic, apoptotic and hypertrophic effects	[114]
	Ischemic Stroke	Activation of vascular cell apoptosis, and vasoconstriction	[115]
	Chronic Heart Disease (CHD)	Increased vasoconstriction and sodium retention leads to hypertension which promotes the progression of CHD	[116]
	Myocardial Infarction	Cardiac fibrosis, collagen synthesis and modified growth of cardiac fibroblast led to the development of atherosclerosis	[117]
	Obesity and dyslipidemia	Upregulation of lipid synthesis through AT II receptors and downregulation of lipolysis through AT I receptors	[118,48]
	Depression	Increased plasma level of AGT produces increased corticotrophin releasing factor which stimulates the hypothalamic-pituitary-adrenal, regulation of norepinephrine, increased release of cytokines, and inhibition of neurogenesis process	[119,120]
AGT G-217A	Essential Hypertension	Vasoconstriction and increased vascular resistance	[16,121,122]
	Hypoxia acclimatization	Increased oxygen saturation	[123]
AGT A-6G	Pre-eclampsia	Vasoconstriction and dysregulation of salt and water homeostasis	[124]
	Obesity	Increased expression of AGT in adipose tissues which is thought to be associated with the development of these tissues. The metabolic alteration may also occur	[125]
	Liver Cirrhosis	AGT activates the Hepatic Stellate Cells (HSC) resulting in increasing the expression of Transforming Growth Factor β-1 (TGFβ-1) and Tissue inhibitor of Metallopeptidase 1(TIMP1) and reduces the degradation of collagen which leads to liver cirrhosis	[89,126]
	Ischemic stroke of atherosclerotic	Activation of vascular cell apoptosis, and vasoconstriction	[127]
	Heart failure	Modification in vascular structure, hemodynamic disturbances and left ventricular hypertrophy	[128]
	Diabetic nephropathy	Sclerosis of the glomerulus, increased cellular growth and proliferation leading to renal damage	[129]
AGT A-20C	Essential hypertension	Vasoconstriction and increased vascular resistance	[80,130]
	Decline in renal function	Reduction in glomerular filtration rate due to vasoconstriction and altered systolic and diastolic pressure	[131]
	Liver Cirrhosis	AGT activates the Hepatic Stellate Cells (HSC) resulting in increasing the expression of Transforming Growth Factor β-1 (TGFβ-1) and Tissue inhibitor of Metallopeptidase 1(TIMP1) and reduces the degradation of collagen which leads to liver cirrhosis	[89,125]
AGT G152A	Coronary artery disease	Vasoconstriction, increased resistance in a coronary artery, the proliferation of myocardial cells, and stimulation of the sympathetic nervous system.	[132]
